# Constraints upon Functionals of the 1-Matrix, Universal Properties of Natural Orbitals, and the Fallacy of the Collins “Conjecture”

**DOI:** 10.1021/acs.jpclett.3c03118

**Published:** 2024-01-29

**Authors:** Jerzy Cioslowski, Krzysztof Strasburger

**Affiliations:** † Institute of Physics, University of Szczecin, Wielkopolska 15, 70-451 Szczecin, Poland; ‡ Max-Planck-Institut für Physik komplexer Systeme, Nöthnitzer Straße 38, 01187 Dresden, Germany; § Department of Physical and Quantum Chemistry, Faculty of Chemistry, Wrocław University of Science and Technology, Wybrzeże Wyspiańskiego 27, 50-370 Wrocław, Poland

## Abstract

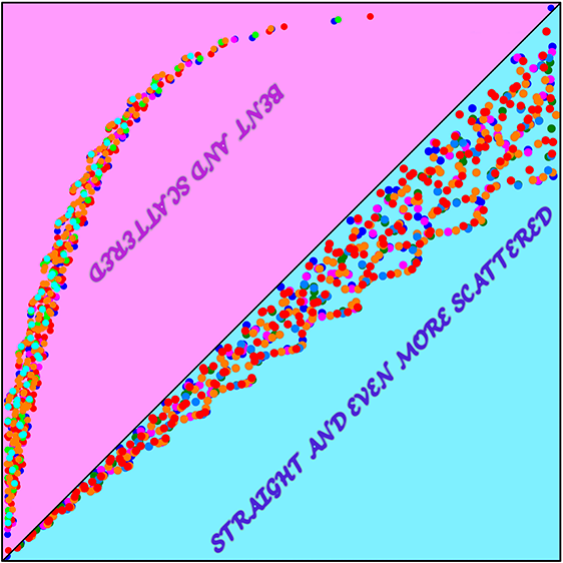

Reliability of quantum-chemical calculations based upon the density functional theory and its 1-matrix counterpart hinges upon minimizing the extent of empirical parameterization in the approximate energy expressions of these formalisms while imposing as many rigorous constraints upon them as possible. The recently uncovered universal properties of the natural orbitals facilitate the construction of such constraints for the 1-matrix functionals. The benefits of their employment in the validation of these functionals are vividly demonstrated by a critical review of the three incarnations of the so-called Collins conjecture. Although the incorporation of rigorous definitions of the correlation energy and entropy, and the identification of individual potential energy hypersurfaces as probable domains of its applicability turn the originally published unsubstantiated claim into a proper conjecture, the resulting formalism is found to be merely a conduit for incorporation of static correlation effects in electronic structure calculations that is unlikely to allow attaining chemical accuracy.

The adjective “complex” describes two distinct characteristics of a (nonrelativistic) electronic wave function Ψ ≡ Ψ(***x***_1_, ..., ***x***_*N*_)^1^ that differ vastly in their importance to the art and science of quantum-chemical calculations. Whereas neither computation nor interpretation of Ψ is significantly affected by its *complex-valuedness* (which in any case comes into play only in the presence of external magnetic fields), they are both severely impacted by its *complexity*. The alleviation of this impact, which has been the target of the century-long efforts of chemists, physicists, and mathematicians, can be attained by either replacing Ψ with its reduced-complexity equivalents or constructing it from less complex quantities.

Within the first of these two approaches, the functional for the electronic energy *E*[Ψ] = ⟨Ψ|*Ĥ*|Ψ⟩, where *Ĥ* is the electronic Hamiltonian, is replaced by its analogue involving a contraction bilinear in Ψ such as, to list the most popular choices,^2^ the two-electron reduced density matrix (the 2-matrix)
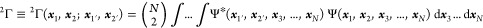
1the one-electron reduced density matrix (the 1-matrix)
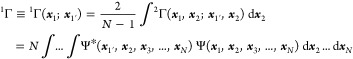
2or the one-electron density
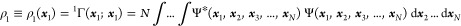
3

The obvious advantage of such an approach is the *N*-independent complexity of ^2^Γ, ^1^Γ, and ρ_1_. However, this simplification carries a heavy price of the respective functionals being far more convoluted than *E*[Ψ]. In the case of *E*[^2^Γ], this means an explicit functional whose domain is extremely complicated due to the requirement of the *N*-representability of ^2^Γ.^3^ Consequently, rigorous electronic structure calculations based upon *E*[^2^Γ] turn out in practice to be computationally as expensive as those invoking *E*[Ψ].^4^ Although the functional *E*[^1^Γ] is not explicit, its unknown part is much smaller in magnitude than that of *E*[ρ_1_]. On the other hand, the *N*-representability conditions for ρ_1_ are trivial,^5−7^ whereas those for ^1^Γ are less so.^8^ The relatively low cost of calculations employing *E*[^1^Γ] and *E*[ρ_1_] comes at the expense of lost rigor as these functionals require empirical parameterization that, especially in the case of *E*[ρ_1_], is usually detrimental to their overall reliability (which should not be confused with the smallness of the average error).^9^ To put it in a nutshell: the complexity of the Ψ equivalent + the complexity of the energy functional that takes it as the argument = the computational effort + the severity of parameterization = const — like everywhere else, there is no free lunch in quantum chemistry!^10,11^

The other approach to the complexity mitigation involves expressing the *N*-electron Ψ in terms of a (in general infinite) sum of products of quantities that depend on fewer than *N* coordinates {***x***_*k*_}. Although many variants of this tensor decomposition are possible^12^ (including those based upon geminals^13−17^), the vast majority of electronic structure calculations employs its most extreme form
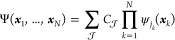
4where , that carries it all the way down to the orthonormal one-electron eigenfunctions (commonly known as spinorbitals) {ψ_*j*_} ≡ {ψ_*j*_(***x***_1_)} of the *ŝ*_*z*_(σ_1_) operator. This representation of Ψ, which is just the FCI (full configuration interaction) expansion written in terms of the Hartree products rather than the Slater determinants, is exact provided that {ψ_*j*_} is a complete set.^18^ In practice, however, only a finite number *M* of the spinorbitals can be included, resulting in an approximate (square-normalized) *N*-electron wave function Ψ̅ ≡ Ψ̅(***x***_1_, ..., ***x***_*N*_) given by a linear combination of  Hartree products. The coefficients  of this linear combination are not fully independent as they have to reflect the constraints imposed upon Ψ̅ by its permutational antisymmetry, the expectation values of the spin operators *Ŝ*^2^ and *Ŝ*_*z*_, and the point group symmetry (if present). The incorporation of other fundamental properties of the exact *N*-electron wave function Ψ into its approximate counterpart Ψ̅ is far less trivial. Thus, the nodal structure of Ψ (which has unexpected features even in the deceptively simple case of the helium atom^19,20^) can be reproduced, depending on the electronic state, quite easily^21^ or with considerable difficulty,^22^ whereas the electron-electron coalescence cusps^23^ cannot be reproduced at all.

From the practical standpoint, the convergence of the approximate expectation value ⟨O̅⟩ ≡ ⟨Ψ̅|*Ô*|Ψ̅⟩, where *Ô* involves one- and/or two-electron operators, to its exact counterpart ⟨*O*⟩ ≡ ⟨Ψ|*Ô*|Ψ⟩, is of great importance to the electronic structure theory. In the case of variational approaches (such as FCI), in which the coefficients  are determined by minimization of ⟨*H̅*⟩ subject to the aforementioned constraints, this convergence is obviously most rapid for the electronic energy. Even so, the decay of the truncation error *ΔE*(*N*, *M*) = ⟨*H̅*⟩ – ⟨*H*⟩ with *M* turns out to be quite slow as indicated by its *M* → ∞ asymptotic lower bound being of the order of .^24^

The dependence of both the accuracy of Ψ̅ and the magnitudes of the optimal  on the one-electron functions constituting the basis set {ψ_*j*_}_*j*=1_^*M*^ (which is a subset of {ψ_*j*_}) brings to the forefront two distinct but intimately related problems of identifying the spinorbitals that, for a given Ψ and *M*, produce (a) the most accurate Ψ̅ and (b) the largest number of  with small magnitudes. The introduction of the natural spinorbitals {ϕ_*n*_} ≡ {ϕ_*n*_(***x***_1_)} that, together with their respective occupation numbers {ν_*n*_} (ordered nonascendingly), enter the Schmidt decomposition
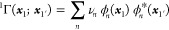
5of the 1-matrix, has been prompted by their promise of providing significant insights into the solution of these twin problems. Unfortunately, this promise, embodied in the rather vague claim that “the introduction of natural spinorbitals leads then instead to a configurational expansion of most rapid convergence”^25^ has not withstood the test of time despite the early enthusiasm brought forth by the encouraging results of limited numerical testing.^26−28^ In fact, although Ψ̅ constructed from the first *M* natural spinorbitals minimizes for a given *M* the wave function error *ΔΨ*(*N*, *M*) = [∫...∫ |Ψ̅(***x***_1_, ..., ***x***_*N*_) – Ψ(***x***_1_, ..., ***x***_*N*_)|^2^ d***x***_1_...d***x***_*N*_]^1/2^ = [2 – (⟨Ψ|Ψ̅⟩ + ⟨Ψ̅|Ψ⟩)]^1/2^ in the case of *N* = 2,^29^ it does not minimize *ΔE*(2, *M*).^24^ Even worse, such a choice of *M* spinorbitals minimizes neither *ΔΨ*(*N*, *M*)^30^ nor *ΔE*(*N*, *M*)^31^ for any *N* > 2. In addition, although the employment of the natural spinorbitals drastically reduces (from ∼*M*^2^ to ∼*M*) the number of the nonzero coefficients among  in the truncated FCI expansions of wave functions describing two-electron systems,^29^ it is not optimal for *N* > 2.^31^ In light of these observations, it is quite surprising that the original claim^25^ remains generally accepted.^32^ On the other hand, it should be emphasized that the accuracy gains and/or the reductions of computational effort realized upon replacing the natural spinorbitals with their optimal counterparts are marginal in comparison with those observed upon analogous replacement involving, e.g., the Hartree-Fock spinorbitals.^33^

Although the initial interest in the natural spinorbitals and their spatial components {φ_*n*_} ≡ {φ_*n*_(*r⃗*_1_)}, known as natural orbitals (NOs), has substantially waned in the wake of the aforementioned studies, the recent years have witnessed a renaissance of research on these one-particle functions spurred by both a growing realization of their usefulness in theoretical nuclear physics^34−36^ and the developments concerning the one-electron reduced density matrix functional theory (1-RDMFT).^37^ Indeed, the substantial progress in the understanding of the properties of the *E*[^1^Γ] functional has been greatly facilitated by the adoption of its equivalent *E*[{ϕ_*n*_}, {ν_*n*_}] that stems from the decomposition 5. This is so because the ensemble *N*-representability of ^1^Γ is assured upon the satisfaction of the trivial constraints ∀_*n*_ 0 ≤ ν_*n*_ ≤ 1 and ∑_*n*_ ν_*n*_ = *N*.^38^ Although proper minimization of *E*[^1^Γ] requires^8^ maintaining the pure *N*-representability of ^1^Γ through additional enforcement of the generalized Pauli constraints (GPCs),^39^ distinguishing between these two domains of *E*[^1^Γ] rapidly becomes irrelevant with increasing *N* and *M*.^40^

Elucidation of the structure of *E*[^1^Γ] is furthered by examination of the constraints imposed upon this functional by certain universal properties of the NOs. Although some of these properties, such as the presence of cusps at the positions of nuclei and the long-range exponential decay, have been uncovered soon after the introduction of the concept of the NOs,^2^ most of them have come to light only very recently thanks to the discovery of the fifth-order off-diagonal cusp in ^1^Γ^41^ that gives rise to the zero-energy Schrödinger equation satisfied asymptotically by both φ_*n*_(*r⃗*_1_) and ν_*n*_ at the limit of *n* → ∞.^42^ The availability of this equation has opened an avenue to facile derivation (which otherwise is quite complicated^43−45^) of various asymptotic power laws, e.g.
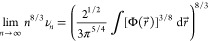
6where Φ(*r⃗*) = ∫∫ ^2^Γ(***x***_1_, ***x***_2_; ***x***_1_, ***x***_2_) δ(*r⃗*_1_ – *r⃗*) δ(*r⃗*_2_ – *r⃗*) d***x***_1_ d***x***_2_ is the on-top two-electron density and the NOs corresponding to different spin components are counted separately (Figure 1). Similar power laws and the *n* → ∞ asymptotics of φ_*n*_(*r⃗*_1_) are obtained for non-Coulombic systems such as the contactium^46^ and the anyons.^47^ Generalizations of the NOs,^48^ such as the energy natural orbitals^49−51^ and the natural transition^52^/ binatural orbitals^53^ are amenable to an analogous treatment. Among them, those involving the eigenfunctions and eigenvalues of the one-electron kinetic energy density matrices (24) and (42,54) are of particular interest as they yield asymptotic expressions that account for the (rather irregular) ∼*n*^2/3^ growth of the  expectation value with *n* (Figure 2). Even more importantly, these expressions lead to the aforementioned estimate of  for *ΔE*(*N*, *M*).^24^

**Figure 1:**
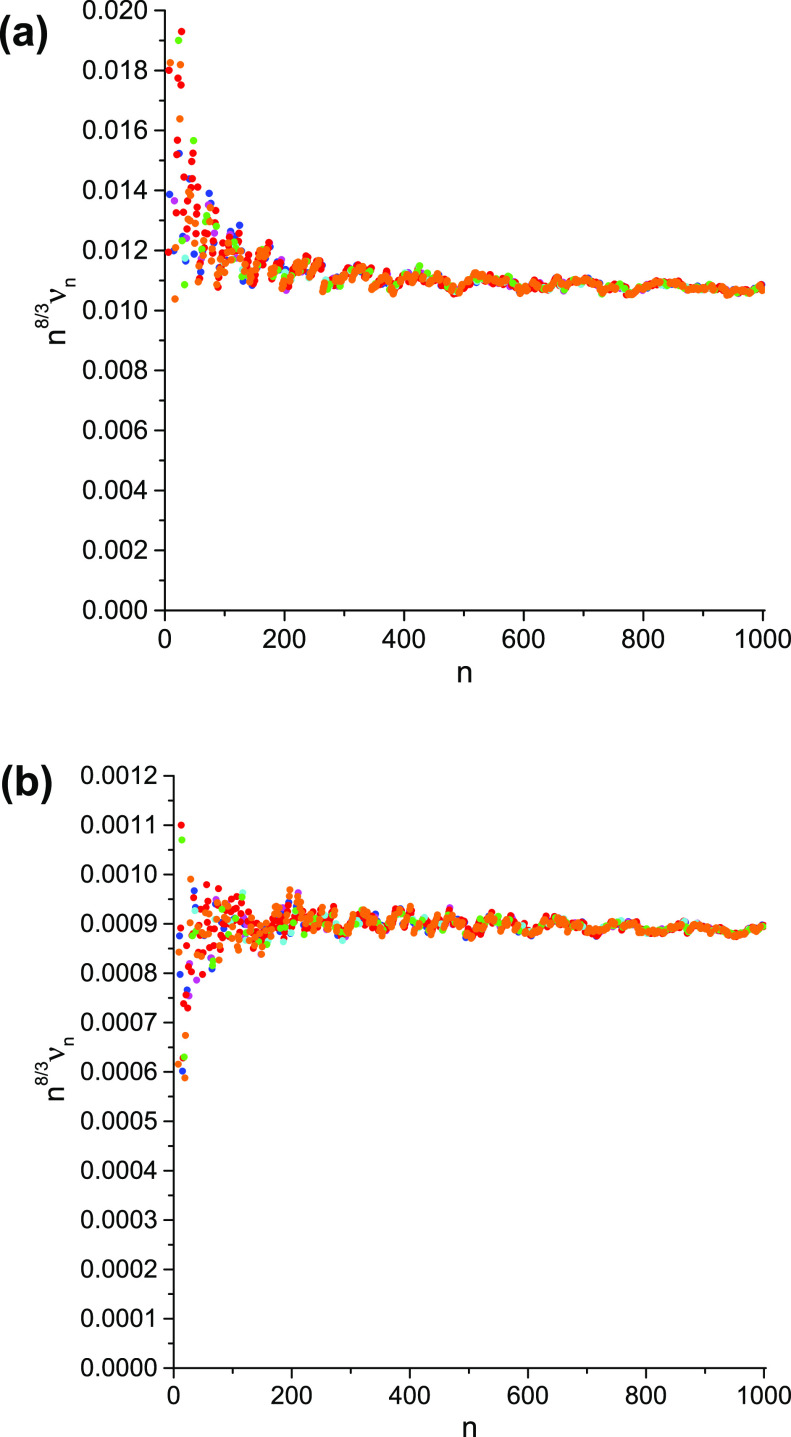
An example of the large-*n* behavior of ν_*n*_: *n*^8/3^ ν_*n*_ vs *n* for the 1-matrices of the ground states of the H_3_^+^ molecular cation at the equilateral geometries with the internuclear distances (a) 1.65 [au] and (b) 6.0 [au]. The blue, magenta, red, cyan, green, and orange dots denote the data pertaining to the NOs with, respectively, the A_1_^′^, A_2_^′^, E′, A_1_^″^, A_2_^″^, and E″ symmetries; identical NOs with two different spin components are counted only once.

**Figure 2:**
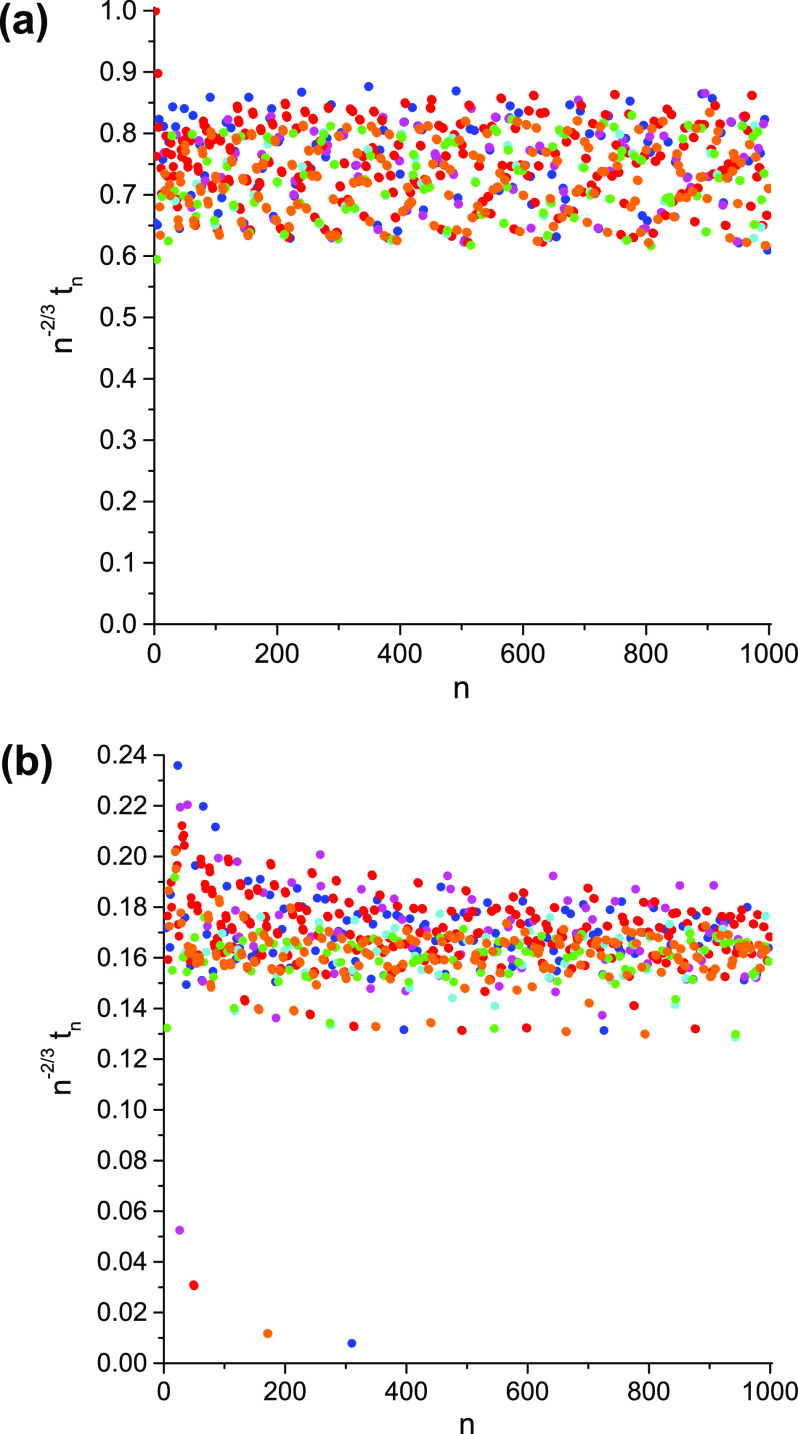
An example of the large-*n* behavior of *t*_*n*_: *n*^–2/3^*t*_*n*_ vs *n* for the 1-matrices of the ground states of the H_3_^+^ molecular cation at the equilateral geometries with the internuclear distances (a) 1.65 [au] and (b) 6.0 [au]. See Figure 1 for the color coding.

It is this estimate, which is equally applicable to truncated FCI expansions and the functionals *E*[^1^Γ] of the 1-matrices constructed from finite numbers of natural (spin)orbitals, that enters the considerations concerning the potential advantages (or the lack thereof) of 1-RDMFT over the wave function-based methods of quantum chemistry. The point of departure for these considerations is the partitioning
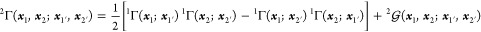
7where
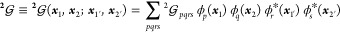
8is the two-electron density cumulant (the 2-cumulant),^55,56^ that allows splitting *E*[^1^Γ] into two terms, namely, the explicit *E*_HF_[^1^Γ] (where HF stands for Hartree-Fock) and the unknown correlation component *U*[^1^Γ] of the electron-electron repulsion energy (also known as the intrinsic correlation energy^57^). The first of these terms, which is system-specific, reads
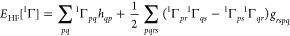
9where ^1^Γ_*pq*_ = δ_*pq*_ν_*p*_ (which follows from the Schmidt decomposition 5), , and *g*_*rspq*_ =  |*r⃗*_1_ – *r⃗*_2_|^–1^  The second term, which is universal, involves the constrained search^58^
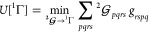
10over all the 2-cumulant tensors  yielding *N*-representable  that conform to the partial trace relations^59,60^
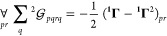
11and
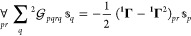
12where ^**1**^**Γ** ≡ {^1^Γ_*pq*_} and the elements of the vector  are given by . In addition, for electronic states that are the eigenstates of the *Ŝ*^2^ and *Ŝ*_*z*_ operators with the eigenvalues of  and , respectively, the elements of these tensors have to satisfy the constraint^59^
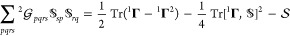
13in which the one-electron spin-flip operator  [equal to 2 *ŝ*_*x*_(σ_1_)] appears in the elements of the matrix  given by .

The elements of the minimizing 2-cumulant tensor  of the constrained search 10 are given by .^59^ Both the corresponding , which follows from eq 8, and ^1^Γ derive from the same electronic wave function Ψ[^1^Γ]. Therefore, for the 1-matrix that minimizes *E*[^1^Γ] = *E*_HF_[^1^Γ] + *U*[^1^Γ], Ψ[^1^Γ] is the ground-state electronic wave function (within the manifold of those with a given spin multiplicity) and  is the corresponding ground-state 2-cumulant. The ground-state 2-matrix that results from the substitution of this 2-cumulant into eq 7 yields in turn the ground-state on-top two-electron density that enters the condition 6 for the occupation numbers pertaining to the ground-state 1-matrix. Consequently, any genuine 1-RDMFT formalism, whether exact or approximate, produces the 2-matrix that allows calculation of any two-electron ground-state property. Moreover, such a formalism yields the pairs ^1^Γ /  that conform to not only the one-index contractions 11 and 12 but also to the identity  =  and the condition 6.

It transpires from eqs 10–13 that evaluation of *U*[^1^Γ] requires computation of the sum , where the tensor  is an unknown function of the vector , the matrices ^**1**^**Γ** and , and the tensor **g** ≡ {*g*_*rspq*_} of the electron-electron repulsion integrals. It should be emphasized that, except in the cases of spin-unpolarized systems () and those with maximum spin polarization (), both  and  have to be included in the argument list of . Moreover, neglecting the dependence of  on **g** gives rise to specious paradoxes exhibited by spurious “algebraic functionals”^61−64^ unless the system in question is an ensemble of noninteracting one- and two-electron subsystems.

The computational cost of evaluating the electronic energy within a given quantum-chemical formalism involving *M* spinorbitals grows like *M*^κ^ (the FCI approach being an exception). It is therefore convenient to introduce the concept of the numerical rank κ as the means for categorizing approximations to *E*[^1^Γ] according to their computational efficiency. With a diagonal ^**1**^**Γ**, only the two-electron integrals {*J*_*pq*_} ≡ {*g*_*pqpq*_} and {*K*_*pq*_} ≡ {*g*_*qppq*_} enter the second sum in the r.h.s. of eq 9, allowing its evaluation from precomputed {*g*_*rspq*_} with ∼*M*^2^ operations. Therefore, the HF-like *E*_HF_[^1^Γ] is a numerical rank-2 functional. The desire to retain this rank upon the addition of *U*[^1^Γ] and thus to keep the 1-RDMFT electronic structure calculations affordable for large systems, has been the driving force behind the development of the so-called *JK*-only functionals,^65^ in which the constrained search 10 is circumvented with the approximation
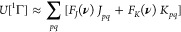
14where *F*_*J*_(**ν**) and *F*_*K*_(**ν**) are some (more or less) empirical functions of the vector **ν** ≡ {ν_*p*_}.^66^ Despite their computational attractiveness, the *JK*-only functionals are fundamentally flawed due to their failure to reproduce the correct behavior of *U*[^1^Γ] at the weak-correlation limit^67^ and the unphysical nature of the constraints imposed upon *U*[^1^Γ] by the approximation 14.^59^ Nevertheless, in light of the above discussion, such functionals are compatible with the genuine 1-RDMFT formalisms as long as they produce 1-matrices whose eigenvalues (i.e., the occupation numbers) satisfy the condition 6.

These observations prompt one to contemplate the possibility of numerical rank-0 and rank-1 approximations to *U*[^1^Γ]. At the first glance, development of such approximations appears to make little sense due to the predominance of the computational cost associated with the evaluation of *E*_HF_[^1^Γ] in the resulting *E*[^1^Γ]. Nevertheless, studies of the functionals belonging to both of these classes have been published. As the numerical rank-0 expressions cannot involve any summation over the indices of spinorbitals, they admit *U*[^1^Γ] ≈ *G*(Tr ^**1**^**Γ**) as the only possible dependence of the approximate *U*[^1^Γ] on its argument. Indeed, the function *G*(*N*) = *c* (*N* – 1), where −0.045 < *c* < −0.030, has been found^68^ to crudely estimate the correlation energy, calculated according to the definition^69^
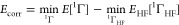
15Although not investigated thus far, an analogous linear regression between *U*[^1^Γ] and *N* = Tr ^**1**^**Γ** is expected to exist.

The story behind the numerical rank-1 functionals is considerably more convoluted. It commences with “a conjecture” published by Collins in 1993.^70^ Supported with neither rigorous theoretical arguments nor a single piece of numerical evidence, and lacking any characterization of its domain of applicability, the claim that *E*_corr_ is proportional to the Jaynes entropy^71^*S*_J_ = −∑_*p*_ ν_*p*_ ln ν_*p*_ computed from the occupation numbers pertaining to the ground-state 1-matrix is obviously a speculative statement rather than a conjecture. Nevertheless, publication of this claim has spurred several numerical studies^72−77^ that, despite some assertions to the contrary, have not confirmed its validity. In particular, the linear regressions between the truncation errors in *E*_corr_ and *S*_J_ that arise from the employment of diverse basis sets in conjunction with approximate electron-correlation formalisms in calculations on the members of the lithium isoelectronic series^72^ and various small molecules^73^ simply reflect the regular reduction of these errors upon the application of increasingly higher levels of theory. However, they do not provide any numerical evidence for the proportionality between the *E*_corr_ and *S*_J_ of different electronic systems. Indeed, in the case of a homogeneous electron gas, the ratio  has been found to vary significantly with the gas density.^74^ Yet another study, involving the members of the helium isoelectronic series and the two-electron harmonium atoms with varying confinement strengths, has uncovered the simultaneous weak-correlation limits of *E*_corr_ → const and *S*_J_ → 0 that are irreconcilable with the original claim.^75^ In the cases of the H_2_^76,77^ and N_2_^77^ molecules, rough linear regressions between *E*_corr_ and *S*_J_ have been found to hold only for internuclear distances close to their equilibrium values.

Turning Collins’ claim into a proper conjecture requires (1) replacing *E*_corr_ with the intrinsic correlation energy *U*[^1^Γ], (2) replacing the Jaynes entropy *S*_J_ with a quantity *S*_nf_[^1^Γ] compatible with the particle-hole equivalence,^78^ and (3) defining the necessary conditions for a linear dependence of *U*[^1^Γ] on *S*_nf_[^1^Γ]. The first of these requirements is trivial to implement, whereas the second one is readily fulfilled by the adoption of the nonfreeness^79^ (a.k.a. “the particle-hole symmetric correlation entropy”^80^)
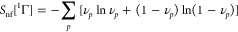
16as the correct measure of “the correlation entropy”. The suitability of these two definitions is supported by the plots of *U*[^1^Γ] against *S*_J_(81) or *S*_nf_[^1^Γ]^82^ that produce straight lines for the H_2_, O_2_, and H_2_O molecules at different geometries (note, however, that the nonzero intercepts of these lines imply a linear dependence rather than a direct proportionality). On the basis of these limited numerical results, one is tempted to formulate “the modified Collins conjecture” that, for a given set {*Z*_*K*_} of nuclear charges, the equality
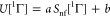
17where *a* and *b* are constants depending on {*Z*_*K*_} and N, and ^1^Γ is the ground-state 1-matrix, approximately holds for all the sets of the nuclear positions {*R⃗*_*K*_}, i.e., for all the points on a given ground-state potential energy hypersurface. However, this conjecture is easily refuted by the following simple *Gedankenexperiment*.

Consider an atom or a molecule  in the ground electronic state whose ionization energy is greater than that of the hydrogen atom. The modified Collins conjecture posits that the equality 17 approximately holds for all the systems composed of  (at arbitrary geometry) and an arbitrary number of protons located at arbitrary positions. Consider two cases: (1) all the protons located at infinity, i.e., at infinite distances from both  and each other, and (2) some of these protons merged with some of the nuclei of . Since the protons at infinity contribute to neither *U*[^1^Γ] nor *S*_nf_[^1^Γ], one concludes that the validity of the modified Collins conjecture on the entire ground-state potential energy hypersurface of some system implies its validity with *the same* constants *a* and *b* on the entire ground-state potential energy hypersurfaces of all the systems isoelectronic with it.

Next, apply the above conclusion to  being the helium atom. Per simple arguments of the perturbation theory,^67^ the intrinsic correlation energy *U*[^1^Γ] of a member of the helium isoelectronic series with the nuclear charge *Z* tends to a constant at the limit of *Z* → ∞, whereas the leading terms in the analogous asymptotics of the deviations of the occupation numbers from their uncorrelated values of 0 and 1 are proportional to *Z*^–2^, implying *Z*^–2^ ln *Z* as the leading asymptotics of *S*_nf_[^1^Γ] at *Z* → ∞. Thus, the modified Collins conjecture fails.

Similarly, consider  being the B^+^ cation (note that the ionization energy of the beryllium atom is smaller than that the hydrogen atom). In this case, the leading term of the large-*Z* asymptotics of *U*[^1^Γ] is proportional to *Z*. As there are certain NOs with occupation numbers tending to constants at *Z* → ∞ (because of the *s*/*p* asymptotic degeneracy),^83^*S*_nf_[^1^Γ] goes to a constant at that limit. The modified Collins conjecture fails again.

These failures bring back the domain-of-applicability question that can be conclusively answered only with rigorous analytic considerations. Due to its definition invoking the constrained search, *U*[^1^Γ] is not readily amenable to the analysis of its purported linear dependence on *S*_nf_[^1^Γ]. However, such an analysis can be carried out with ease for the responses of these two quantities to one-electron perturbations. Thus, the first-order response of the ground-state electronic energy to the perturbation λ∑_*k*=1_^*N*^ χ̂(*r⃗*_*k*_) involving a one-electron operator with the matrix elements {χ_*qp*_} = {⟨ϕ_*q*_(***x***_1_)| χ̂(*r⃗*_1_)|ϕ_*p*_(***x***_1_)⟩} is *λ E*^χ^[^1^Γ], where *E*^χ^[^1^Γ] = ∑_*pq*_^1^Γ_*pq*_ χ_*qp*_ by virtue of the Hellmann-Feynman theorem^84−87^ (here and in the following, the standard notation , etc., is used). On the other hand,
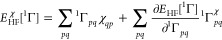
18as *E*_HF_[^1^Γ] is not stationary with respect to variations in ^1^Γ. Consequently,
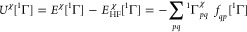
19where (see eq 9)
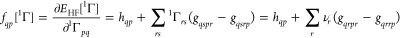
20is the matrix element of the generalized Fock operator *f̂*. Moreover,
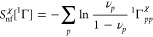
21Thus, for the equality 17 to hold not only at a given point of the ground-state potential energy hypersurface but also in its vicinity (and/or also in the presence of, e.g., electric field), *U*^χ^[^1^Γ] has to be proportional to *S*_nf_^χ^[^1^Γ] for all the perturbations *λ χ̂*. In other words, the condition
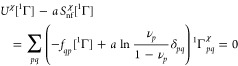
22has to be satisfied for *all*^**1**^**Γ**^**χ**^ ≡ {^1^Γ_*pq*_^χ^} such that Tr ^**1**^**Γ**^**χ**^ = 0. Needless to say, its equivalent
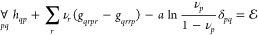
23where  is a constant, is not satisfied by the natural spinorbitals and their occupation numbers. Consequently, eq 17 is not valid even locally. However, eq 22 may hold for *a particular*^**1**^**Γ**^**χ**^ despite the violation of condition 23. This observation may explain the approximate linear dependence of *U*[^1^Γ] on *S*_nf_[^1^Γ] observed for certain nuclear motions.^81,82^ On the other hand, one-electron properties (e.g., multipole moments) computed as respective derivatives of the energy functional *E*_HF_[^1^Γ] + *a S*_nf_[^1^Γ] + *b* with respect to the perturbation strength λ are expected to be inaccurate.

In all of the above considerations, the 1-matrix that enters *E*_HF_[^1^Γ], *U*[^1^Γ], and *S*_nf_[^1^Γ] is the ground-state ^1^Γ that minimizes *E*[^1^Γ] = *E*_HF_[^1^Γ] + *U*[^1^Γ]. As the computational cost of evaluating *S*_nf_[^1^Γ] is dominated by that of procuring this ^1^Γ (or at least an accurate approximation of it), the practical importance of such a formulation of the Collins conjecture is nil. In order to address this issue, the minimization of the functional *Ẽ*[^1^Γ] = *E*_HF_[^1^Γ] + *a S*_nf_[^1^Γ] + *b* has been proposed as the means for computing approximate ground-state electronic energies.^82,88^ However, such an approach is riddled with inconsistencies. First of all, even if one assumes that the equality *U*[^1^Γ] = *a S*_nf_[^1^Γ] + *b* holds for *any ground-state* 1-matrix, its validity for *arbitrary*^1^Γ cannot be ascertained. This observation, coupled with the *de facto* limited domain of applicability of eq 17 even for the ground-state 1-matrices, makes the derivation of eq 23, which relies on *arbitrary* variations of ^1^Γ, mathematically suspect. Second, the lack of the dependence of *a S*_nf_[^1^Γ] + *b* on **g** gives rise to a ^1^Γ /  paradox that concerns the first-order response of the ground-state electronic energy to a two-electron perturbation. Per the Hellmann-Feynman theorem,^84−87^ this response involves the 2-matrix derived from the minimizing 2-cumulant . In the case of *Ẽ*[^1^Γ], this 2-matrix turns out to be given by the partitioning 7 with a vanishing 2-cumulant, which violates the sum rules 11 and 12 unless ^1^Γ is idempotent. This paradox can be resolved only by either setting the coefficient *a* to zero (which yields the Hartree-Fock energy shifted by a constant *b*) or making *a* and/or *b***g**-dependent.

The minimizer ^1^Γ̃ of *Ẽ*[^1^Γ] is obtained by solving the system of equations 23 with **ν**, ***h***, and ***g*** replaced by their counterparts **ν̃**, ***h*~**, and ***g*~** pertaining to ^1^Γ̃ rather than to ^1^Γ. The *p* = *q* component of this system implies a linear relationship between the expectation values {*f*_*pp*_[^1^Γ̃]} of the generalized Fock operator and , which rules out ^1^Γ̃ being the 1-matrix of a system of correlated electrons (Figure 3). This is so because, as already mentioned in this Letter, such a system is characterized by the large-*p* asymptotics of ν_*p*_ ∼ *p*^–8/3^ and *t*_*p*_ ∼ *p*^2/3^, the latter carrying over to {*f*_*pp*_[^1^Γ̃]} due to the predominance of the kinetic energy term. Although the same objection can be raised about the Hartree-Fock approximation, it should be emphasized that, in contrast to the *Ẽ*[^1^Γ] functional, which originates from the fiat approximation 17 and is claimed to include electron correlation effects, *Ẽ*_HF_[^1^Γ] derives from a well-defined variational wave function that is Coulombically uncorrelated by default.

**Figure 3:**
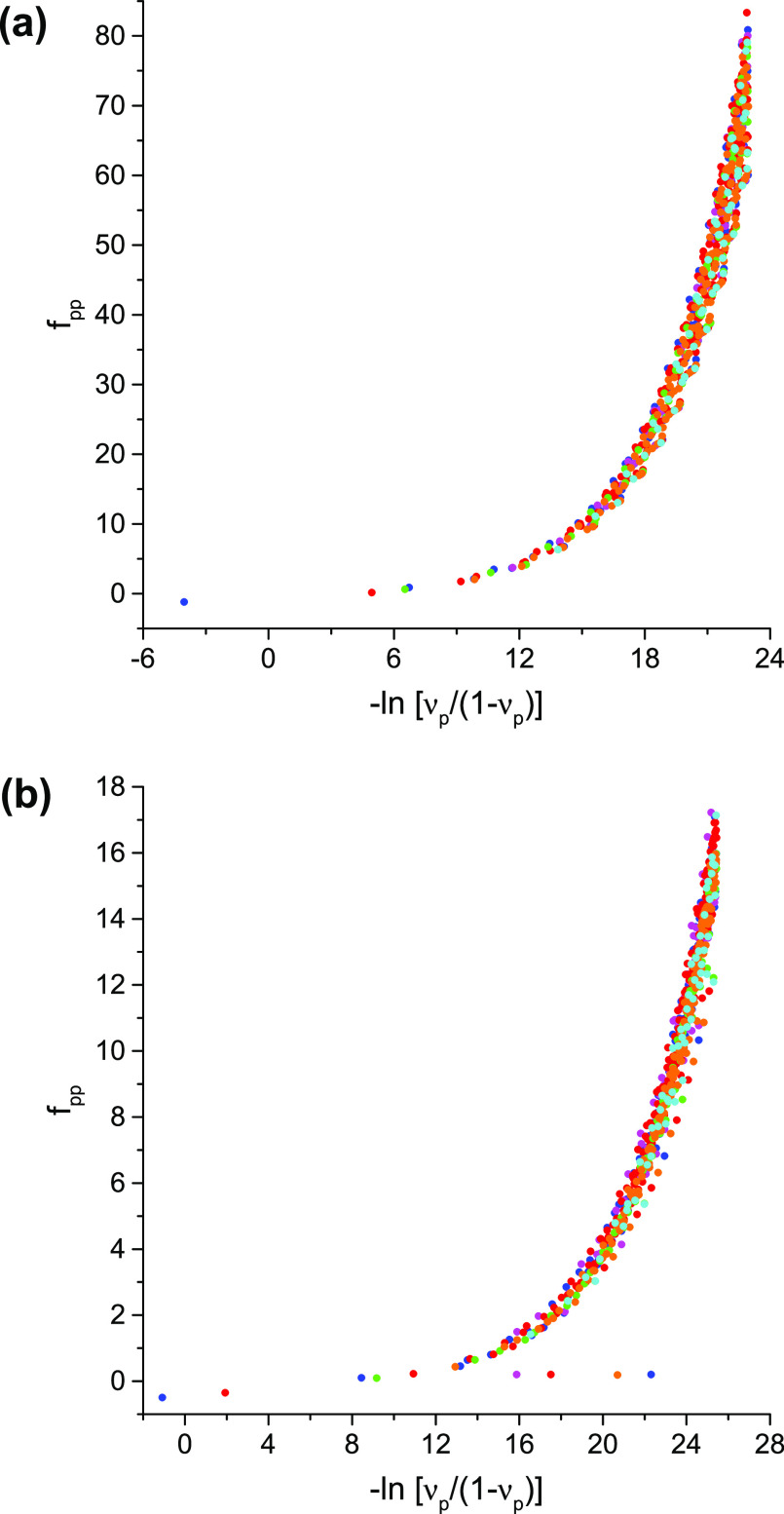
An example of the incompatibility of the 1-matrix with the condition 23: *f*_*pp*_ vs  for the 1-matrices of the ground states of the H_3_^+^ molecular cation at the equilateral geometries with the internuclear distances (a) 1.65 [au] and (b) 6.0 [au]. See Figure 1 for the color coding.

In light of these observations, it is justified to view the formalism that invokes the minimization of *Ẽ*[^1^Γ] as just a semiempirical one-particle theory employing spinorbitals and their occupation numbers as auxiliary quantities. Regarding it as a variant of 1-RDMT^82^ is not warranted.^89^

One may wonder whether employing some other numerical rank-1 functional *D*[^1^Γ] in place of *S*_nf_[^1^Γ] would provide a better approximation to *U*[^1^Γ] that, while not disposing of the aforedescribed inconsistencies, would nevertheless result in an improved conformance of ^1^Γ̃ with the large-*p* asymptotics of ν_*p*_ and *f*_*pp*_. Since such a replacement would turn eq 23 into
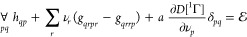
24the functional *D*[^1^Γ] should conform to the large-*p* scaling of . Imposing the particle-hole equivalence upon this asymptotics yields
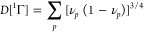
25as a possible candidate for the replacement functional. However, although the overall dependence of *f*_*pp*_ on  is indeed linear, the correlation between these two quantities remains poor (Figure 4). Even worse, repetition of the aforepresented *Gedankenexperiment* produces the leading large-*Z* asymptotics of ∼*Z*^–3/2^ and ∼1 for *U*[^1^Γ] of the members of the helium and beryllium isoelectronic series, respectively. This recurring failure underscores the necessity of incorporating some dependence on **g** in the constants *a* and/or *b* that, in addition to eliminating the inconsistencies, would provide “the missing powers” of *Z* in these asymptotics. Some efforts in this direction have been recently reported.^90^ The attempts at improving the performance of approximate formulations of the density functional theory by incorporating add-ons analogous to *S*_nf_[^1^Γ] and *D*[^1^Γ]^91−93^ should also be mentioned in this context. However, the resulting energy expressions are not size consistent and thus are of doubtful value in practical quantum-chemical calculations.

**Figure 4:**
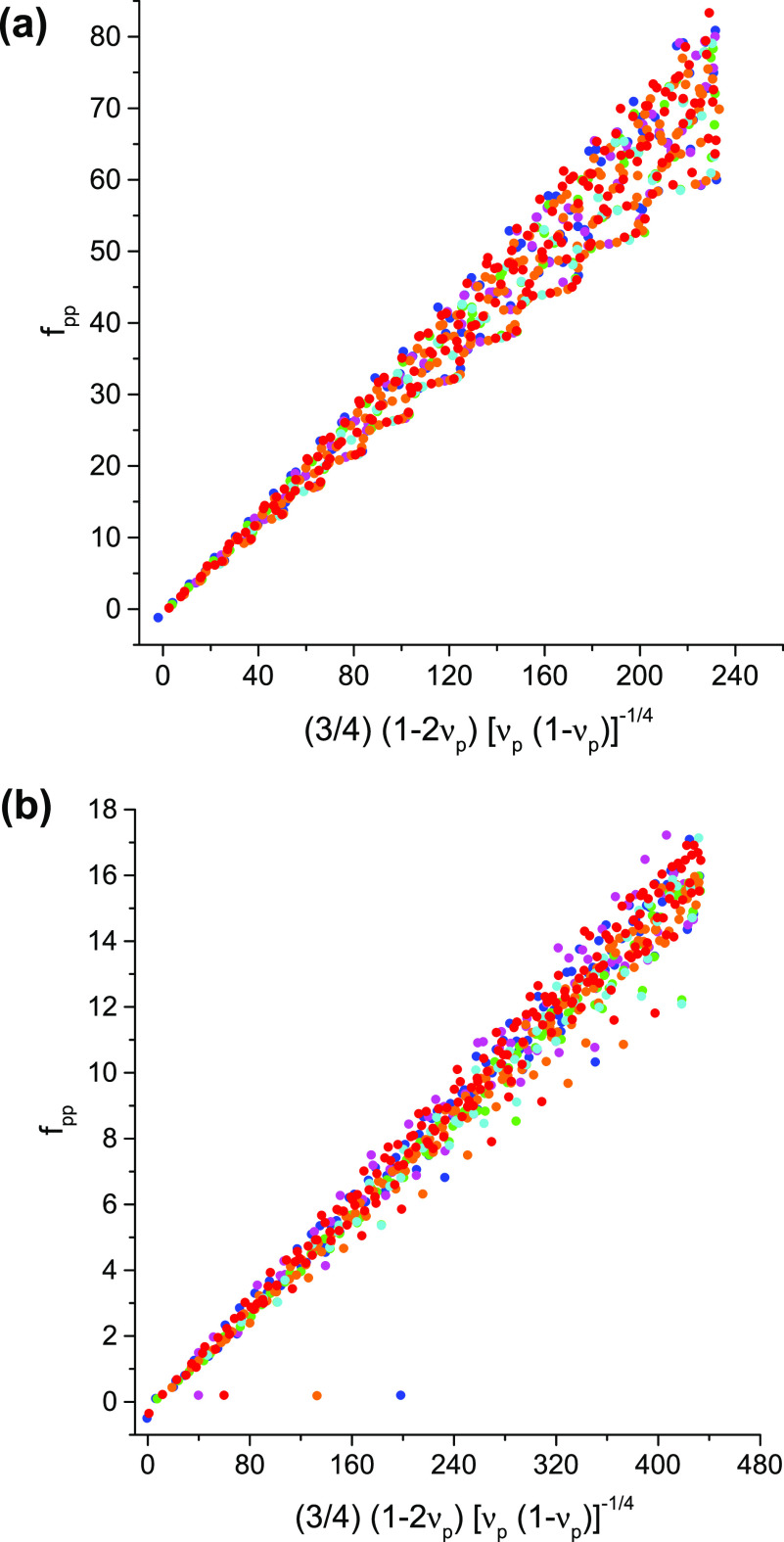
An example of the improved compatibility of the 1-matrix with the condition 24: *f*_*pp*_ vs  for the 1-matrices of the ground states of the H_3_^+^ molecular cation at the equilateral geometries with the internuclear distances (a) 1.65 [au] and (b) 6.0 [au]. See Figure 1 for the color coding.

Reliability of quantum-chemical calculations based upon the density functional theory (DFT) and its 1-matrix analogue (1-RDMFT) hinges upon limiting to the absolute minimum the extent of empirical parameterization in the approximate energy expressions of these formalisms while imposing as many rigorous constraints upon them as possible. Until recently, the constraints pertaining to the 1-matrix functionals have been far less numerous than those employed in the development of their one-electron density counterparts. However, since the former functionals are usually formulated in terms of the natural orbitals, bringing the recently uncovered universal properties of these quantities into play permits reduction of this disparity. This development is an example of novel applications of the natural orbitals that, despite failing to deliver upon the initial promise of maximizing the accuracy of approximate wave functions, remain among the most important concepts of the electronic structure theory.

The benefits of employing a broad instrumentarium of analytical and numerical tools in the validation of the 1-matrix functionals are vividly demonstrated by the insights gained with the critical review of the three incarnations of the so-called Collins conjecture presented in this Letter. The first of these incarnations, published in the form of an unsubstantiated claim^70^ unsupported by numerical evidence,^72−77^ has been later turned into a proper conjecture with the help of rigorous definitions of the correlation energy^57^ and entropy,^79^ and the identification of individual potential energy hypersurfaces as probable domains of its applicability.^81,82^ However, this second incarnation, called “the modified Collins conjecture” in this Letter, does not withstand theoretical scrutiny, invoking a simple helium/beryllium *Gedankenexperiment*. In addition, its tentative domain of applicability is ruled out by arguments based upon the first-order response theory. The observation that neither of these incarnations is of any practical importance has given rise to the idea of deploying the modified Collins conjecture in conjunction with a variational formalism. However, the resulting third incarnation, which inherits the flaws with its predecessors, cannot be regarded as a variant of the 1-RDMFT formalism. Attempts at amelioration of these difficulties by replacing the nonfreeness with another correlation strength measure compatible with the universal properties of the natural orbitals produce functionals that again fail the *Gedankenexperiment*. These failures clearly demonstrate that the numerical rank-1 functionals are merely conduits for incorporation (in a crude manner) of static correlation effects in electronic structure calculations that is unlikely to allow attaining chemical accuracy.^94^

A note on computational details: the data displayed in Figures 1–4 have been derived from electronic wave functions described elsewhere.^24^
